# How patients experience thyroid eye disease

**DOI:** 10.3389/fendo.2023.1283374

**Published:** 2023-11-09

**Authors:** Terry J. Smith, Laszlo Hegedüs, Ira Lesser, Petros Perros, Kimberly Dorris, Michele Kinrade, Patti Troy-Ott, Laura Wuerth, Mukund Nori

**Affiliations:** ^1^ Kellogg Eye Center, Department of Ophthalmology and Visual Sciences and Department of Internal Medicine, University of Michigan Medical School, Ann Arbor, MI, United States; ^2^ Department of Endocrinology, Odense University Hospital, Odense, Denmark; ^3^ Department of Psychiatry, Harbor-UCLA Medical Center, Torrance, CA, United States; ^4^ Institute of Translational and Clinical Research, Newcastle University, Newcastle upon Tyne, United Kingdom; ^5^ Graves’ Disease and Thyroid Foundation, Rancho Santa Fe, CA, United States; ^6^ Scientific Solutions, RareLife Solutions, Inc., Westport, CT, United States

**Keywords:** thyroid eye disease, Graves’ orbitopathy, thyroid-associated ophthalmopathy, patient survey, patient experience, quality of life, active disease, chronic disease

## Abstract

**Objective:**

To determine the impact of thyroid eye disease (TED) on patients in various stages of the disease.

**Background:**

TED is a debilitating and potentially sight-threatening inflammatory autoimmune disease that is frequently misdiagnosed. Challenging quality-of-life (QoL) issues can persist long after the active phase of disease has subsided.

**Methods:**

A 62-question survey was designed as a hypothesis-generating instrument to identify key issues confronting patients ≥18 years old with physician-diagnosed TED. Questions focused primarily on physical and emotional status, and QoL experiences in the 2 months prior to the survey. Data for individual questions are presented as summary statistics. Correlations between questions were determined using χ^2^ analyses.

**Results:**

The 443 respondents were 18 to >80 years old; >90% female, and >80% from the United States. Time since TED diagnosis ranged from <1 year to >10 years. Participants provided >500 free-form responses describing experiences of living with TED. Physical signs/symptoms were experienced by 307/443 (69%) patients. Of those responding to the QoL questions (N = 394), 53 (13%) reported symptoms improving, 73 (19%) reported symptoms worsening, and 255 (65%) reported no change in the 2 months prior to the survey. The most bothersome signs/symptoms were dry/gritty eyes, light sensitivity, bulging eyes, and pressure or pain behind the eyes. Respondents <60 years were significantly (p < 0.0001) more likely to report symptomatic TED than older patients. Of 394 respondents, 179 (45%) reported feeling depressed and/or anxious, 174 (44%) reported concern about their appearance, and 73 (19%) avoided public situations; 192 (49%) reported declines in confidence or feelings of general well-being, and 78 (20%) reported an inability to achieve goals. Activities limited by TED included reading, driving, and socializing. The proportion of respondents experiencing these negative QoL measures was higher when patients reported experiencing >5 symptoms, had been diagnosed within the last 5 years, or were <60 years of age.

**Conclusions:**

Physical manifestations of TED impact QoL for patients through all phases of the disease. It is essential that physicians and healthcare professionals become more familiar with patient experiences such as those described here to better help patients manage their disease.

## Introduction

Thyroid eye disease (TED), also known as thyroid-associated ophthalmopathy, and Graves’ orbitopathy (GO)/ophthalmopathy, is a debilitating, inflammatory autoimmune disease that is disfiguring and potentially sight-threatening (1–3). TED is a relatively rare disease with a calculated prevalence between 90 and 250/100,000 (1, 4). It occurs in 25%–40% of patients with Graves’ disease (GD) as a consequence of loss of immune tolerance to the thyroid-stimulating hormone and insulin-like growth factor 1 (IGF-I) receptors (1, 2). Despite the majority of patients with GD presenting at some point in their disease journey with hyperthyroidism, approximately 10% of patients with TED are found to have normal thyroid hormone levels or to manifest autoimmune (primary) hypothyroidism (5).

TED is a lifelong condition (1, 6). It typically presents with an active or acute phase of inflammation lasting 2-5 years, during which time physical signs and symptoms change/progress, potentially resulting in vision changes (3, 5). Ocular manifestations stabilize during the subsequent inactive, non-progressive phase (3, 5). TED can be classified as mild, moderate-to-severe, or sight-threatening (7, 8). Manifestations of TED can include eyelid retraction, ocular dryness/grittiness, eyelid redness, pain with eye movement, pressure sensation behind the eyes, and excessive tearing (2, 3, 5). Proptosis, diplopia, and vision dysfunction can develop, becoming chronic and persisting for years (3).

Patients with TED can experience significant disease burden, frequently the consequence of suboptimal medical management or treatment (2, 9). TED is associated with excess co-morbidity and mortality, as well as early retirement and loss of productivity (10–14). Decreased quality of life (QoL) results from physical manifestations and their negative impact on mental health (15, 16). The wide-ranging emotional toll of TED frequently includes anxiety and depression, both of which can persist in chronic disease (3, 15, 17, 18). Patients are often faced with incorrect diagnoses, yielding therapeutic delays, all too often resulting from healthcare provider unfamiliarity with TED (2, 19–21).

Relatively little recent evidence informs the current experiences, perspectives, and QoL of individuals living with TED (9, 16, 20, 22). An overarching question to be considered was the degree to which disease-related reductions in QoL widely attributed to early, active TED might become less problematic after the transition of patients to chronic, non-progressive disease. Further, how do potential differences in disease affect QoL with respect to sex and age? Many earlier patient surveys have employed tools including the TED-QOL and the GO-QOL to evaluate overall QoL (23). However, these surveys, including the validated ThyPRO instrument, are very structured and are not designed to capture individual patient experiences in free-text format (22, 24–28). Very few surveys report in-depth patient interviews probing patient medical condition and level of well-being (20, 22).

Physicians and patients may have complementary views on TED and QoL (29). Individualized treatment requires that healthcare providers understand the needs of their patients. The goal of our study was to determine the impact of TED on patients along their disease journey, from its development into chronic disease. Because of the rapidly evolving therapeutic landscape for this relatively rare disease, we believe that establishing a contemporary baseline of patient perceptions, disease impact, and treatment options will be useful as new therapeutic options continue to emerge. In this paper, we report results generated from an online survey that explored personal experiences of patients with TED and their access to management/therapeutic options.

## Methods

A 62-question, hypothesis-generating survey was developed to explore the patient experience of TED. We constructed and administered an exploratory survey to identify key issues confronting patients with TED. The questions asked focused mainly on patient experiences during the 2-month interval prior to their taking the survey. This should have minimized any imprecision stemming from imperfect recall. Included were questions concerning disease history, current physical and emotional status, physical signs (as determined by physicians and described by the patient), symptoms, and treatments received (**Supplemental Table 1**). The survey included free-form questions allowing respondents to describe their personal experiences. We collected responses from patients aged ≥18 years with physician-diagnosed TED. Responses from a pilot survey were used to refine the final survey, which was publicly available June 2022–July 2022 using SurveyMonkey. The survey was provided in English and was publicized as being accessible through the Graves’ Disease and Thyroid Foundation (GDATF) website (https://gdatf.org/) and the oneGRAVESvoice community platform (https://www.onegravesvoice.com/). We promoted the survey on social media platforms, including Facebook, Instagram, Twitter, LinkedIn, and through paid Google search advertisements.

Respondents were required to grant permission for the use of their de-identified, respondent-aggregated data for survey participation. Data are anonymized in accordance with General Data Protection Regulations (30). To ensure patient privacy, we redacted any details from narrative responses that could allow identification of individual respondents, including location and treatment center. Data for individual questions are presented as summary statistics and are calculated from the number of responses received for each question. Correlations between the answers to various questions were determined based on χ^2^ analyses using GraphPad Prism for macOS v10.0.0.

## Results

### Respondent demographics and TED-related characteristics

#### Overview of respondents

**Figure 1:**
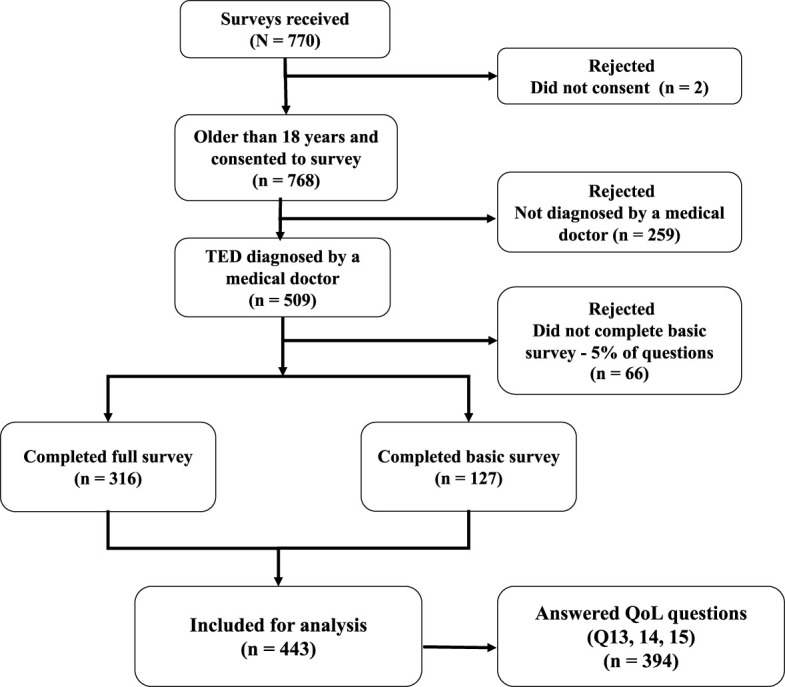
Respondent disposition for analysis. QoL, quality of life; TED, thyroid eye disease.

Of the 770 people who opened the survey, 509 (66%) met participation criteria of being ≥18 years of age and having a physician-rendered diagnosis of TED. A total of 443 patients responded to the core questions (first 11 questions) at a minimum. This level of response was considered sufficient to be included in parts of the final analyses (**Figure 1**). QoL questions were completed by 394 respondents, and the entire survey was completed by 316 patients. More than one-half of the respondents (229/443; 52%) received their initial diagnosis of TED by an ophthalmologist, whereas 137/443 (31%) were originally diagnosed by an endocrinologist. The TED diagnosis was eventually confirmed by an ophthalmologist for 371/443 (84%) of patients.

#### Age and demographics of respondents

The age of respondents ranged from 18 to >80 years, and 231/443 (52%) were between the ages of 50 and <70 years (**Table 1**). Most respondents (401/443; 91%) were female and >80% were from the United States and self-identified as White or Caucasian.

**Table 1:** Patient demographics.

Patient characteristics N = 443		Responses n (%)
Age, years	18-29	13 (2.93)
	30-39	28 (6.32)
	40-49	78 (17.61)
	50-59	106 (23.93)
	60-69	125 (28.22)
	70-79	75 (16.93)
	80-89	16 (3.61)
	90 or older	0 (0)
	Prefer not to disclose (but I am age 18 or older)	2 (0.45)
Sex	Female	401 (90.52)
	Male	37 (8.35)
	Nonbinary	1 (0.23)
	Prefer not to disclose	4 (0.90)
Nationality	United States	365 (82.39)
	Canada	25 (5.64)
	United Kingdom	17 (3.84)
	Australia	6 (1.35)
	Israel	3 (0.68)
	Other	27 (6.09)
Ethnicity	Asian or Pacific Islander	15 (3.39)
	Black or African American	17 (3.84)
	Hispanic or Latino	19 (4.29)
	Multiracial or Biracial	4 (0.90)
	Native American or Alaskan Native	2 (0.45)
	White/Caucasian	366 (82.62)
	Do not wish to disclose	14 (3.16)
	Other	6 (1.35)
Diagnosing physician	Ophthalmologist	229 (51.69)
	Optometrist	24 (5.42)
	Endocrinologist	137 (30.93)
	Primary care physician	37 (8.35)
	Other	16 (3.61)

#### Obtaining a correct diagnosis

**Figure 2:**
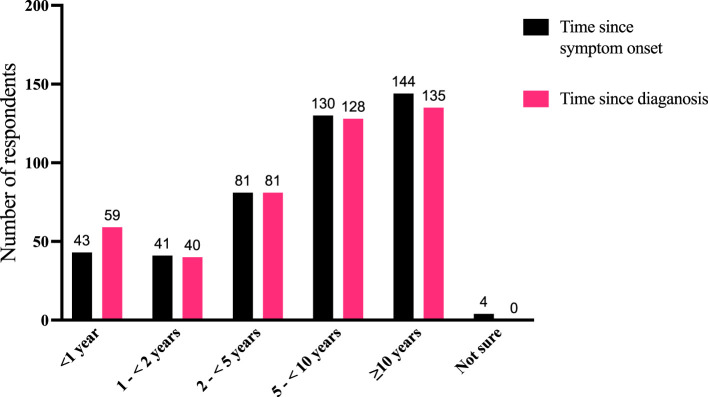
Comparison of time periods for disease duration as measured by the time since symptom onset (“How long ago did you first experience symptoms of TED?”) and time since diagnosis (“How long ago were you diagnosed with TED?”). Fifty-eight individuals reported that their symptoms started before diagnosis, 20 reported symptom onset was after diagnosis, and 361 reported both symptom onset and diagnosis occurred within the same time window (4 respondents were not sure when their symptoms began). N = 443 responses. TED, thyroid eye disease.

Patients reported that the time elapsing from their TED diagnosis or the time since they first experienced TED symptoms to the time of the survey ranged from <1 year to >10 years (**Figure 2**). Out of 443 respondents, 180 (41%) had been diagnosed within <5 years, 128 (29%) were diagnosed between 5 to <10 years, and 135 (30%) had been diagnosed ≥10 years earlier. Some patients described difficulties encountered in their diagnostic journey (**Supplemental Table 2**):

- *“It was hard getting a diagnosis. I visited 2 local doctors who just prescribed eye drops that did nothing. It wasn’t until I went to a major teaching hospital in a large city that I received a correct diagnosis.”*
- *“I saw 5 different doctors. My first eye doctor told me my complaints were mental – all in my head. This was 3 months prior to finding a provider that listened and understood.”*
- *“It also took many months and many doctor visits to get an accurate diagnosis, which is demoralizing and terribly frustrating.”*

#### Current perception of TED symptoms

The majority of respondents (307/443; 69%) reported currently experiencing symptoms of TED, whereas 91/443 (21%) were not, and 45/443 (10%) were unsure (**Table 2**).

**Table 2:** Disease characteristics.

Currently experiencing symptoms of thyroid eye disease N = 443	Responses n (%)
Yes	307 (69.30)
No	91 (20.54)
Not sure	45 (10.16)
Disease trajectory during the past 2 months n = 394	
Getting worse	26 (6.60)
Getting a little worse	47 (11.93)
Staying about the same	255 (64.72)
Getting a little better	24 (6.09)
Getting better	29 (7.36)
Not sure	13 (3.30)
Disease phase n = 394	
Active	94 (23.86)
Not active	202 (51.27)
Not sure	98 (24.87)

Almost one-quarter of the respondents (94/394; 24%) reported that their disease was in the active phase (continuing to change or getting worse), whereas 202/394 (51%) reported that their disease has not recently changed (inactive/chronic phase), and 98/394 (25%) indicated uncertainty. These answers were congruent with the question as to the trajectory of their TED signs/symptoms during the most recent 2 months: 255/394 (65%) reported unchanged signs/symptoms, whereas 53/394 (13%) said that their signs/symptoms were improving, 73/394 (19%) reported that they were worsening, and 13/394 (3%) were not sure.

Respondents reported dry/gritty eye, light sensitivity, bulging eyes, and pressure or pain behind the eye as the most burdensome ocular symptoms in the previous 2 months (**Table 3**, **Supplemental Table 3**).

- *“At [60+] years old and having TED for 40 years, I am still uncomfortable with the bulging eyes, uneven eyelids after surgery, sensitivity to light, redness, and general discomfort.”*
- *“Eyes do not look straight ahead, comfortable eye position for me after TED is much lower than neutral so to lift my eye up hurts, driving more than 15 minutes is difficult and then this creates tremendous neck pain and … migraines.”*
- *“When I felt increased pressure at work and home, my symptoms got worse.”*

**Table 3:** Which physical symptoms of thyroid eye disease have bothered you the most (made life more difficult/caused pain or suffering) during the past 2 months? (Select all that apply.)

Symptom (Select all that apply) n = 329	Responses n (%)
Dry/gritty eye	215 (65.35)
Sensitivity to light	185 (56.23)
Bulging eyes	143 (43.47)
Pressure or pain around or behind eye	139 (42.25)
Tearing (too much eye watering)	134 (40.73)
Eye redness	127 (38.60)
Double vision (seeing 2 images instead of 1)	115 (34.95)
Eyelid swelling	108 (32.83)
Blurred/cloudy vision or vision loss	105 (31.91)
Inability to close eyelid	68 (20.67)
Eyes that aim in different directions	53 (16.11)
Color vision loss	7 (2.13)
Other	27 (8.21)
None of the above	21 (6.38)

### Quantitative analyses of respondent demographics and TED-related characteristics

**Figure 3:**
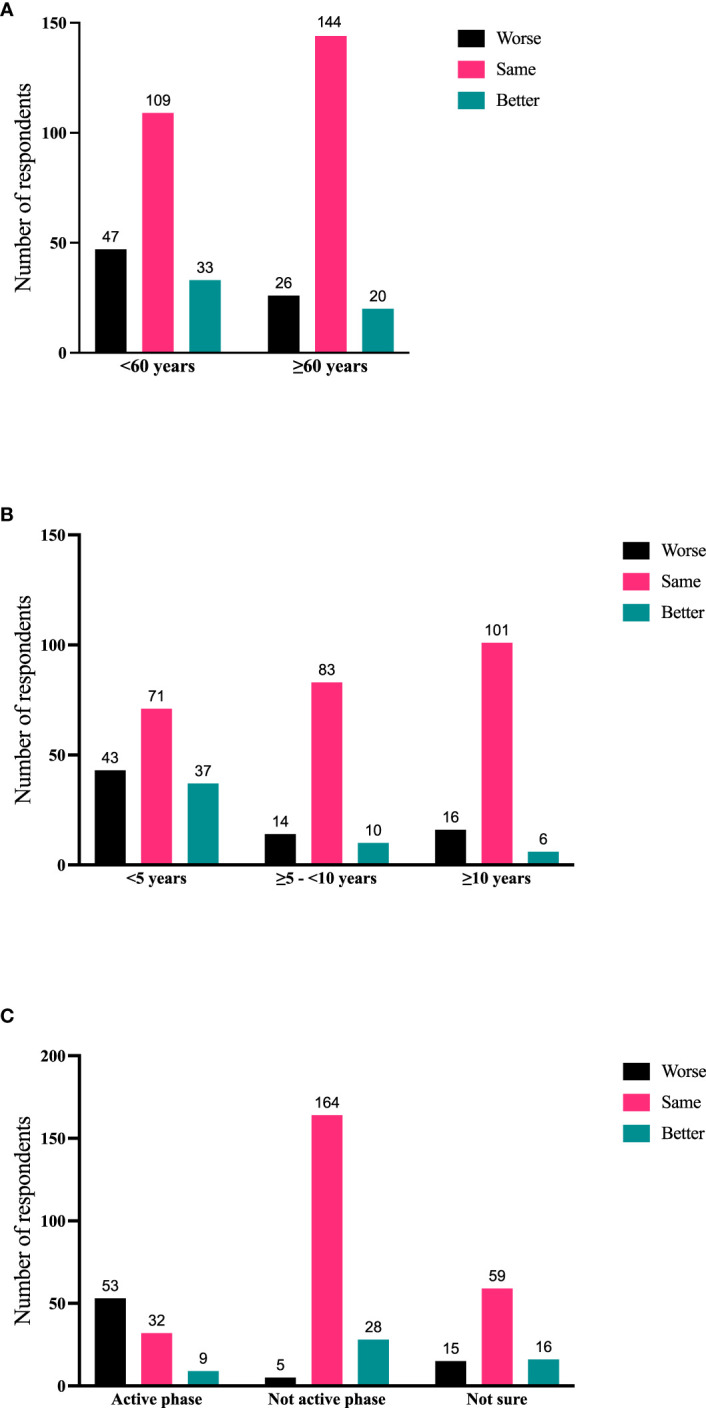
**(A)** Current age of respondent compared with disease trajectory (worse, same, better during the 2 months prior to the survey); N = 379; p = 0.0009. **(B)** Time since diagnosis of TED compared with disease trajectory (worse, same, better during the 2 months prior to the survey); N = 381; p < 0.0001. **(C)** Disease phase (active/inactive/not sure) compared with disease trajectory (worse, same, better during the 2 months prior to the survey). N = 381; p < 0.0001. TED, thyroid eye disease.

Significant correlations emerged between the respondents’ age (**Figure 3A**), time since diagnosis (**Figure 3B**), and disease phase (active or not active, **Figure 3C**) with disease trajectory (worse, same, better) within the previous 2 months. Respondents who report longer-duration TED were less likely to manifest signs/symptoms consistent with active TED phase even if they were experiencing some symptoms. Respondents <60 years of age were significantly (χ^2^ (1) = 17.62; p < 0.0001) more likely to report symptomatic (active) TED (63/145; 43.45%) than those who were older (31/150, 20.67%). Time from TED diagnosis and time from TED sign/symptom development to time of survey participation both correlate with currently experiencing symptoms (p < 0.0001; p = 0.0002), disease phase (active or inactive) (p < 0.0001; p < 0.0001), and trajectory of disease (p < 0.0001; p < 0.0001) (**Table 4**).

**Table 4:** Chi-square analysis of age, time since diagnosis, or symptom onset to disease symptoms, phase, and trajectory.

	Currently experiencing symptoms Number of respondents		Active phase Number of respondents		Disease trajectory Number of respondents		
Age, years	Yes	No	Yes	No	Worse	Same	Better
18-50	80	25	32	43	23	54	25
50-60	76	20	31	39	24	55	8
60-70	86	31	18	71	18	84	10
>70	63	15	13	48	8	60	10
Total	305	91	94	201	73	253	53
	**χ^2^ (3)** ^a^ **= 1.73; p = 0.6309**		**χ^2^ (3) = 17.69; p = 0.0005**		**χ^2^ (6) = 24.64; p = 0.0004**		
Time since diagnosis^b^, years							
<1	55	0	34	3	22	12	12
1-2	31	6	10	13	10	13	12
2-5	54	15	13	39	11	46	13
5-10	71	41	16	65	14	83	10
>10	96	29	21	82	16	101	6
Total	307	91	94	202	73	255	53
	**χ^2^ (4) = 29.28; p < 0.0001**		**χ^2^ (4) = 75.82; p < 0.0001**		**χ^2^ (8) = 73.70; p < 0.0001**		
Time since symptom onset^b^, years							
<1	39	1	28	4	19	8	12
1-2	34	4	14	12	10	13	10
2-5	58	15	13	36	12	46	9
5-10	71	38	17	62	15	81	14
>10	102	33	22	88	16	106	8
Total	304	91	94	202	72	254	53
	**χ^2^ (4) = 21.87; p = 0.0002**		**χ^2^ (4) = 63.19; p < 0.0001**		**χ^2^ (8) = 66.28; p < 0.0001**		

a Number in parentheses = degrees of freedom.

b Time from diagnosis or symptom onset to date of survey. Bold values represent chi-square analyses and corresponding p values.

**Figure 4:**
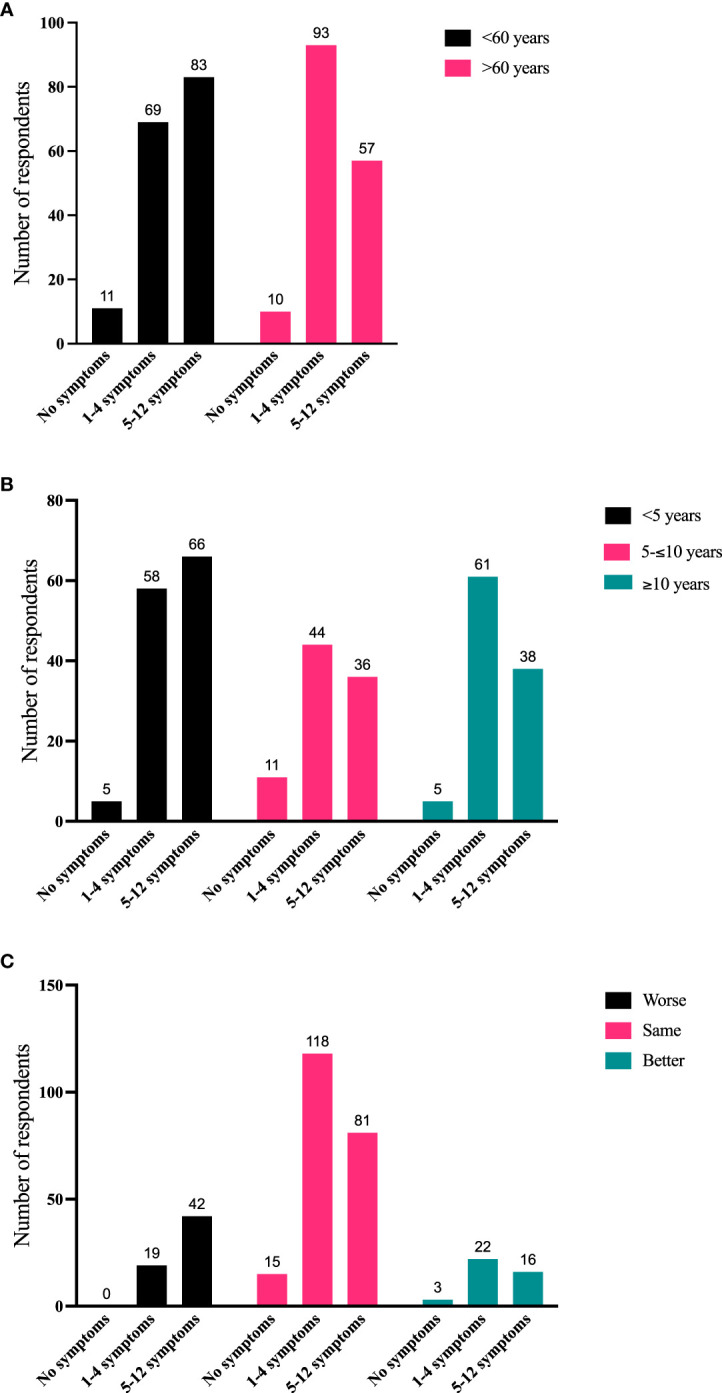
**(A)** Number of symptoms in the last 2 months (grouped as no symptoms, 1-4 symptoms, or 5-12 symptoms) compared with current age (<60 years, >60 years). N = 323; p = 0.015. **(B)** Number of symptoms in the last 2 months compared with time since diagnosis (<5 years, 5-10 years, >10 years). N = 324; p = 0.0198. **(C)** Number of symptoms in the last 2 months compared with disease trajectory in the last 2 months. N = 316 (The number of respondents reporting the combination of no symptoms and disease getting worse was too small for statistical analysis. Therefore, responses to “1-4 symptoms” were compared with “5-12 symptoms”. N = 298; p = 0.0005).

To further quantify TED sign/symptoms experienced during the most recent 2 months, 324 respondents were stratified into the following groups: without symptoms, 1-4 symptoms, and 5-12 symptoms. Significant correlations emerged between number of symptoms compared with age, time since diagnosis, and disease trajectory. Individuals >60 years of age tended to have proportionately fewer symptoms than younger individuals (p = 0.015) (**Figure 4A**). Further, individuals with longer-duration TED had relatively fewer symptoms during the last 2 months (p = 0.0198) (**Figure 4B**). Individuals with 5-12 symptoms more frequently reported their disease as getting worse during the last 2 months (1-4 symptoms vs 5-12 symptoms; p = 0.0005) (**Figure 4C**).

No significant correlations were identified among those currently experiencing symptoms and sex (p = 0.433), race/ethnicity (p = 0.738), or country of residence (USA or other countries; p = 0.728). Further, respondent age did not correlate with current TED symptoms (p = 0.631).

### QoL analysis and narrative responses

This survey posited 3 questions relating directly to psychosocial QoL issues experienced during the 2 months prior to taking the survey: 1) negative feelings and emotions; 2) self-perceived declines in well-being; and 3) self-perceived limitations in activities due to TED (**Supplemental Table 1** Questions 13, 14, and 15).

#### Emotional feelings and declines in well-being affected by TED in the last 2 months

**Figure 5:**
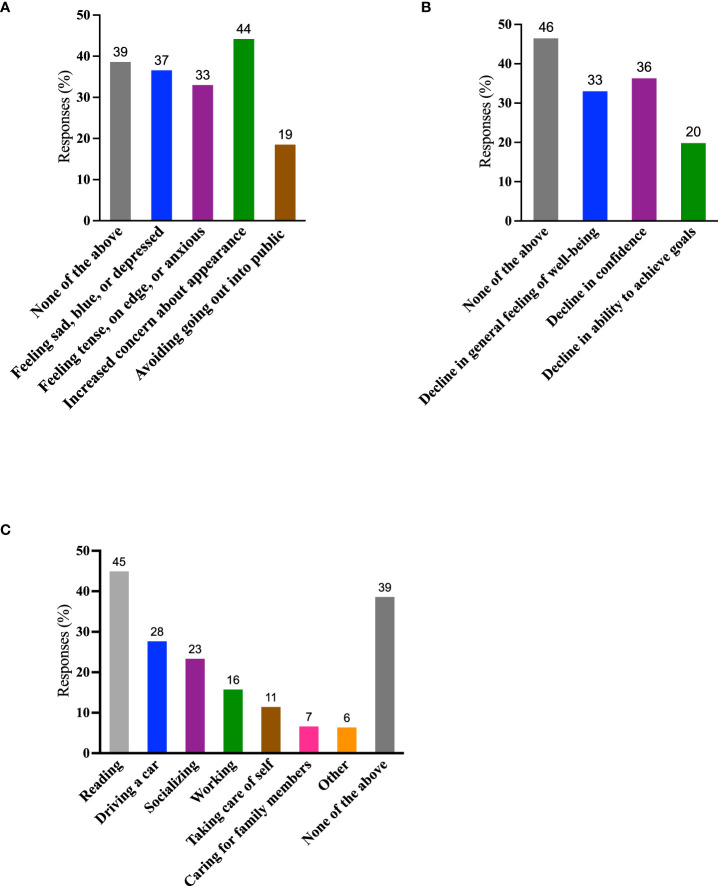
QoL measures. **(A)** During the past 2 months, which of the following have you experienced because of TED? (Negative feelings) (Select all that apply); **(B)** During the past 2 months, which of the following have you experienced because of TED? (Declines) (Select all that apply); **(C)** During the past 2 months, which activities have been limited by TED? (Select all that apply). Data are presented as percentages of the 394 individuals who answered these questions. A total of 130/394 (33%) individuals reported “none of the above” to both questions **(A)** and **(B)** QoL, quality of life; TED, thyroid eye disease.

Individuals with TED frequently experience significant emotional burdens (15, 17). Of the 394 respondents who answered the QoL questions, approximately one-half (179, 45%) of respondents reported feeling depressed and/or anxious, and 174 (44%) expressed concerns about their appearance. Seventy-three (19%) reported avoiding public situations (**Figure 5A**). Of the 242/394 individuals who reported ≥1 negative feeling (sad, blue, or depressed; tense, on edge, or anxious; increased concern about appearance; avoidance of going out into public), 42/242 (17%) reported all 4 negative feelings and an additional 43/242 (18%) reported 3 of 4 negative feelings.

One hundred and ninety-two (49%) respondents reported a decline in confidence and/or in feelings of well-being. Seventy-eight of 394 (20%) experienced a decline in achieving goals (**Figure 5B**). Of the 211/394 individuals who experienced ≥1 decline (decline in confidence, sense of well-being, or ability to achieve goals), 43/211 (20%) reported all 3 negative states and an additional 54/211 (26%) reported 2 of 3.

- *“I hate it. Graves’ disease has robbed me of so much in life mentally, [its] taking a physical toll on my appearance has destroyed my self-image.”*
- *“It is frustrating and defeating.”*

#### Activities limited due to TED

Activities most frequently self-reported as limited during the previous 2 months included reading (177/394; 45%), driving (109/394; 28%), socializing (92/394; 23%), and working at employment (62/394; 16%). In our survey, 242/394 (61%) reported ≥1 activity limited by TED, whereas only 152/394 (39%) reported that their activities were unlimited by the disease (**Figure 5C**). Approximately one-half of the respondents 200/394 (51%) reported a limitation in 1-3 of these activities and 42/394 (11%) reported that 4-6 were limited.

- *“Having TED is more difficult than many people think, and it really puts limitations on doing things I used to enjoy, especially reading.”*
- *“I feel most people do not understand my limitations. My limitations have drastically changed my quality of life.”*

**Figure 6:**
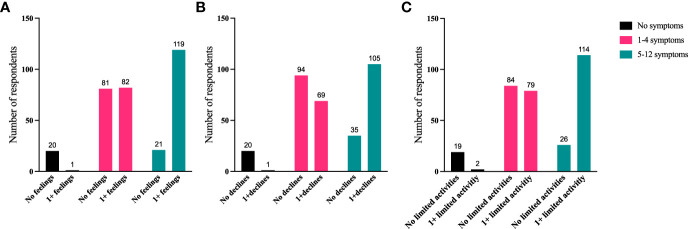
A higher proportion of individuals with ≥5 physical symptoms report reduced QoL compared with those with no or <5 symptoms. QoL measures **(A)** “No negative feelings” or “1 or more negative feelings”, **(B)** “No declines” or “1 or more declines”, **(C)** “No activities limited” or “1 or more activities limited by TED” compared with number of symptoms currently being experienced: “No symptoms”, “1-4 symptoms”, or “5-12 symptoms”. N = 324; p <0.0001. QoL, quality of life; TED, thyroid eye disease.

**Figure 7:**
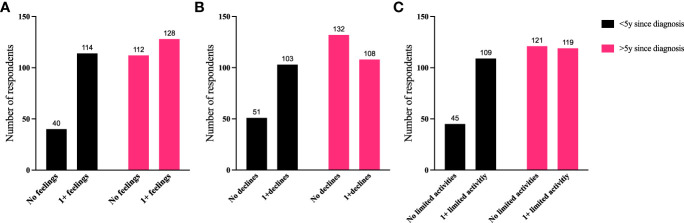
A higher proportion of individuals diagnosed with TED for <5 years report reduced QoL compared with those with longer duration TED. QoL measures **(A)** “No negative feelings” or “1 or more negative feelings”, **(B)** “No declines” or “1 or more declines”, **(C)** “No activities limited” or “1 or more activities limited by TED” compared with time since diagnosis: “<5 years since diagnosis” or “>5 years since diagnosis”. N = 394; p < 0.0001. QoL, quality of life; TED, thyroid eye disease.

**Figure 8:**
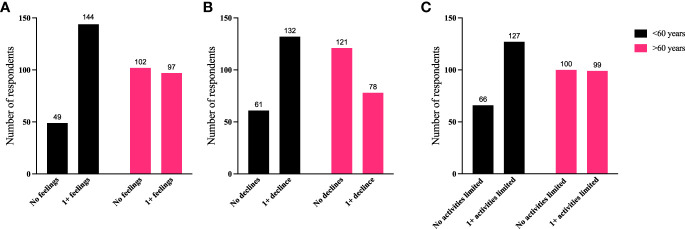
A higher proportion of individuals <60 years old report reduced QoL compared with those >60 years old. QoL measures **(A)** “No negative feelings” or “1 or more negative feelings”, **(B)** “No declines” or “1 or more declines”, **(C)** “No activities limited” or “1 or more activities limited by TED” compared with age of respondent: “<60 years” or “>60 years”. N = 392; p < 0.0001 for feelings and declines and p = 0.0013 for limitations in activity. QoL, quality of life; TED, thyroid eye disease.

**Figure 9:**
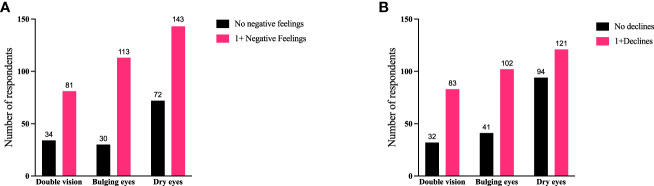
Individuals experiencing double vision or bulging eyes report proportionately more negative feelings and declines compared with individuals with dry/gritty eyes. Specific symptoms of TED (double vision, bulging eyes, or dry/gritty eyes) compared with **(A)** negative feelings or **(B)** declines currently being experienced by respondents. QoL measures are combined as “no negative feelings” and “1 or more negative feelings” or declines. When all 3 symptoms are compared with “no negative feelings” or “1 or more negative feelings”, χ^2^ (2) = 6.609; p = 0.0367. When the 3 symptom groups are compared with “no declines” or “1 or more declines” χ^2^ (2) = 12.24; p = 0.0022. There were no significant correlations between the 3 symptoms and limitations of activities. Specific significant correlations are bulging eyes to dry/gritty eye and “1 or more negative feelings” 113/143 (79%) to 143/215 (67%), respectively (p = 0.0102); bulging eyes to dry/gritty eyes to “1 or more declines” 102/143 (71%) to 121/215 (56%), respectively (p = 0.004); double vision to dry/gritty eye and “1 or more declines” 83/115 (72%) and 121/215 (56%), respectively (p = 0.0046). QoL, quality of life; TED, thyroid eye disease.

To further identify how aspects of TED affect QoL, responses were grouped for analysis (**Figures 6**–**9**). Individuals experiencing physical signs/symptoms also reported significantly more negative QoL measures (negative feelings, declines in well-being, and limitations in activity; p < 0.0001). The proportion of respondents experiencing negative feelings, declines, and limitations was significantly higher for those with >5 symptoms; p < 0.0001 (**Figure 6**). Individuals diagnosed <5 years prior to the survey had a higher proportion of negative QoL measures than those who have experienced TED for longer periods; p < 0.0001 (**Figure 7**). The proportions of negative QoL measures were also higher in individuals <60 years of age compared with those older than 60 years of age; p < 0.0001 for feelings and declines, and p = 0.0013 for limitations in activity (**Figure 8**).

We also compared specific physical signs/symptoms (double vision, bulging eyes, and dry/gritty eyes) with these QoL measures to determine how the seriousness of the symptom might affect patients (**Figure 9**). Individuals experiencing double vision or bulging eyes report proportionately more negative feelings and declines than individuals reporting dry/gritty eyes; however, there were no significant correlations with limitations in activity. Respondents experiencing bulging eyes reported negative feelings in a greater percentage than patients with dry/gritty eyes (113/143 [79%] to 143/215 [67%], respectively; p = 0.0102). Bulging eyes also correlated with ≥1 declines in well-being, confidence, or the ability to achieve goals in a greater percentage than dry/gritty eyes (102/143 [71%] to 121/215 [56%], respectively; p = 0.004). Similarly, double vision correlated with ≥1 declines at a higher percentage than dry/gritty eyes (83/115 [72%] vs 121/215 [56%], respectively; p = 0.0046).

#### Individual perceptions of experiences with TED

Respondents provided personal insights into their experiences using free-form narrative responses describing life with TED (**Supplemental Table 4**).

- *“This is a very difficult disease that causes tremendous damage to one’s health, ability to be independent, and psychologic well-being. As is the case with other autoimmune diseases, it is unpredictable, triggers other, significant health problems, and is always with you.”*
- *“…My confidence has taken a huge knock because of my eyes that are bulging and teary and nose that’s runny.”*
- *“The doctors that I saw, while all highly qualified, almost uniformly discounted the psychological effects of TED. Several bluntly told me that my appearance should be of no concern since sight functionality had been restored after surgery. I was taken aback by this level of callous disregard for patient experience and concerns.”*
- *“My wife and I were dining out at a local restaurant and a young boy (8 or 9) having dinner with his family looked at me, turned to his dad and said… ‘look, dad, that man has scary eyes.’ My wife and I paid our bill and that [family’s] bill as well and left. I [have] never been out to eat in a restaurant since. This was about 5 years ago.”*

## Discussion

The results of this survey provide an overview of patient experiences while living with TED. Responses included self-reported signs/symptoms and narrative comments describing personal experiences and interactions with healthcare providers. Through responses to multiple choice and open-ended questions, patients provided medical histories and their perception of physical and emotional signs and symptoms experienced during a 2-month period (June-July 2022), thereby providing a snapshot of recent experiences. Because most questions captured relatively recent experiences (the 2-month interval prior to study participation) variations in recall should not have substantially affected patient responses.

The entire survey was completed by 316 respondents, and the responses of 443 participants were considered sufficiently complete to be included in parts of the final analyses, since the key questions about demographics were answered. QoL questions were answered by 394 individuals. Respondents demonstrated a high-level commitment to sharing their experiences, struggles, and insights into their disease, as evidenced by the relatively large number of patients completing a long survey (62 questions) and providing >500 free-form responses. This robust engagement is consistent with an earlier report of patients’ eagerness to share their TED experiences during in-depth patient interviews (20). Inclusion of several questions with open fields for free-form responses has enhanced our ability to capture emotions and experiences in the patients’ own words. Representative samples of these narrative responses are included in this manuscript, and the rest of the responses relating to the topics covered in this paper can be found in **Supplemental Tables 2**-**4**.

Several current findings corroborate those of earlier studies. Most respondents (69%) reported currently experiencing signs and symptoms, despite only 24% of them indicating that they were currently within the active phase of their disease (defined as continuing to change or getting worse). This finding, like earlier observations, highlights the underrecognized long-term burden of TED (15, 18). During the 2-month reporting period, signs and symptoms remained about the same for 65% of respondents; in contrast, 13% reported improving, whereas 19% reported worsening. Among the most burdensome symptoms were dry/gritty eyes, sensitivity to light, bulging eyes, and pressure or pain behind the eyes. This troublesome pattern in chronic TED is similar to that previously reported (15, 18).

Our findings are consistent with previous studies reporting delays in receiving the correct diagnosis of TED, ranging from 2-16 months, and eye-related symptoms developing well before the initial physician visit (19, 31–33). Many respondents reported difficulties obtaining the correct diagnosis and/or getting adequate treatment.

Limitations of activities associated with daily life, most frequently including reading, driving, socializing, and employment, are consistent with earlier findings, as are the emotional consequences of TED, including negatively impacted daily functions, facial appearance, depression and/or anxiety, reduced confidence, and feeling of general well-being (15, 20, 34, 35). The emotional burdens imposed by TED in the current study generally align with previous findings, in patients with GD without or with TED (13, 15, 20). Individuals with chronic diseases have been found to manifest reduced QoL with increased frequency, likely a consequence of their particularly emotional responses to physical distress and impairment. In the context of thyroid dysfunction, which can elicit changes in mood and provoke anxiety and depression, individuals with TED may be particularly vulnerable (11, 15). The inclusion of free-form responses to the survey enabled respondents to convey, in their own words, the emotional toll exacted by TED.

A major limitation of this survey was its dependence on the self-identification of respondents as adults with a medical doctor–confirmed TED diagnosis. Other limitations of our survey include its substantial length (posing potential burden for patients with serious visual disturbances), being presented only in the English language, and potentially limited internet access of prospective respondents. Some questions required a respondent to have sufficient knowledge of TED to answer properly.

Our survey provided grouped responses for certain questions, such as respondent age, time since first symptom, and time since physician-rendered diagnosis. These groupings were pragmatically assigned based on known characteristics of TED. This approach limited our ability to finely discriminate between some parameters and our ability to perform quantitative statistics, including multivariate analyses. Choices of cut-offs for larger age subgroups were based on observations from the data, which tended to show distinct changes around age 60 years and a natural transition of disease characteristics between 5-10 years of TED duration.

The survey respondents were in large part White/Caucasian females from the United States. There were no major differences in response when the survey was filtered by sex; however, the numbers of males was relatively low (8%). Clinically meaningful statistical analyses based on country, race, and ethnicity were not possible because of inadequate numbers of respondents from countries outside the United States and races other than White/Caucasian. It is also possible that response rates and some of the negative physical and psychosocial experiences reported in the survey were confounded due to the impact of coronavirus disease 2019 (COVID-19) and its treatments.

Our survey was exploratory and hypothesis-generating by design. We set about identifying important and as yet unresolved issues for further, more detailed investigation. These include exploration of psychosocial aspects of TED in defined ethnic, geographic, and age populations. Impact of more timely TED diagnosis and treatment has been the topic of substantial conjecture but remains to be quantified and specifically related to geographic location and disease outcome (36). Educating at-risk patients, such as those with autoimmune thyroid disease, about the earliest signs and symptoms of TED might alert them to seek timely evaluation by an eye care professional. Thus, better understanding the initial patient journey in TED may well improve our ability to educate patients.

We were able to determine that disease-related reductions in QoL widely associated with early, active TED became less prominent as patients transitioned to chronic, non-progressive disease. We found many QoL measures were proportionately worse in respondents with disease duration of <5 years compared with those with longer duration TED. Importantly, however, many negative responses regarding QoL (>100 per question) came from respondents with TED for >5 years. As would be anticipated, patients who were experiencing symptoms reported more negative QoL measures. This phenomenon was especially true for those experiencing >4 symptoms. Individuals <60 years of age also tended to have proportionately more negative feelings, declines, and limitations to activities compared with older patients.

In a debilitating and disfiguring disease such as TED, measures of treatment success such as the clinical activity score are not necessarily sufficient to determine patient levels of well-being (37). Physicians could consider QoL issues just as valid as functional treatment outcomes when discussing TED with their patients. It has been suggested that QoL evaluations should be part of routine care and should factor more prominently into treatment decisions such as types of surgery (38, 39).

We wanted to examine how QoL measures of relatively easily reversible symptoms like dry/gritty eye compared with more challenging symptoms, such as double vision or bulging eyes. There was a significant correlation between the respondents reporting more negative feelings and declines with bulging eyes compared with dry/gritty eyes. There was also a higher percentage of significant declines reported with double vision compared with dry/gritty eyes, but none of these 3 signs and symptoms specifically correlated with limitations in activity. We might have expected that difficulties such as double vision would have been far more limiting than dry/gritty eyes, which can frequently be treated with eye drops. This is an area that might benefit from further exploration.

Although the sample size of males was relatively small, we found no important sex differences identified in the respondent cohort. It would be useful to explore the psychosocial aspects of TED in greater detail with a more evenly distributed population based on sex as well as race, ethnicity, geography, and various cultures.

Importantly, results from this survey reveal the important, persistent needs of patients with TED. Those needs continue well beyond the transition of the disease from the active to the chronic, non-progressive phase. The results should inform healthcare providers of their critical role in ongoing patient follow-up and periodic re-evaluation.

## Conclusions

The findings reported here reveal a perception among patients that some physicians lack an awareness of TED and fail to recognize the signs, symptoms, and negative impact of this disease on QoL. These shortcomings likely underlie, at least in part, the unmet needs of patients with this disfiguring and potentially sight-threatening disease. The persistence of important and bothersome disease manifestations clearly impacts activities of daily life, regardless of disease duration. It is essential that physicians and other healthcare professionals who are treating TED become more familiar with patient experiences such as those described here. Results from our survey should invigorate the importance of long-term follow-up and management of patients. Their healthcare providers need to better accommodate the needs of these patients and improve their journey with TED.

## Data availability statement

The raw data supporting the conclusions of this article will be made available by the authors, without undue reservation.

## Author contributions

TS: Conceptualization, Methodology, Supervision, Writing – original draft, Writing – review & editing. LH: Conceptualization, Methodology, Writing – original draft, Writing – review & editing. IL: Conceptualization, Methodology, Writing – original draft, Writing – review & editing. PP: Conceptualization, Methodology, Writing – original draft, Writing – review & editing. KD: Conceptualization, Funding acquisition, Writing – review & editing, Methodology. MK: Conceptualization, Data curation, Formal Analysis, Methodology, Writing – original draft, Writing – review & editing. PT: Conceptualization, Methodology, Project administration, Writing – review & editing. LW: Conceptualization, Supervision, Writing – review & editing. MN: Conceptualization, Methodology, Supervision, Writing – original draft, Writing – review & editing.
