# Supplementary material for: How patients experience thyroid eye disease
Source: Front Endocrinol (Lausanne). 2023 Nov 9;14:1283374. doi: 10.3389/fendo.2023.1283374 (PMC10665908; doi:10.3389/fendo.2023.1283374)
Supplement: Supplementary file 1 [file Table_1.docx]

**Supplemental Data**

**SUPPLEMENTAL TABLE 1 Survey questions**

| Q1: By participating, you agree that you are age 18 or older and you consent to use of your responses as part of the aggregated and anonymized results. |
| --- |
| Q2: Was your TED diagnosis made by a medical doctor (MD, DO, or optometrist/OD)? |
| Q3: What kind of doctor diagnosed you with TED? |
| Q4: Was your diagnosis of TED made or confirmed by an ophthalmologist? |
| Q5: How long ago were you diagnosed with TED? |
| Q6: How long ago did you first experience symptoms of TED? |
| Q7: What is your current age? |
| Q8: What is your gender? |
| Q9: In what country do you currently reside? |
| Q10: Which of the following best describes you? (Ethnicity) |
| Q11: Are you currently experiencing symptoms related to TED? |
| Q12: In general, how would you describe your TED symptoms over the past 2 months (compared to the previous 2-month period)? |
| Q13: Over the past 2 months, which of the following have you experienced because of TED? (Select all that apply.) (Feeling sad, blue or depressed; Feeling tense, on edge or anxious; Increased concern about appearance; Avoid going out in public) |
| Q14: Over the past 2 months, which of the following have you experienced because of TED? (Select all that apply.) (Decline in general feeling of well-being; Decline in confidence; Decline in ability to achieve goals) |
| Q15: Over the past 2 months, which activities have been limited by TED? (Select all that apply.) |
| Q16: Is your TED currently in an active phase (that is, continuing to change or get worse)? |
| Q17: If any of your most recent thyroid lab results were considered too high, indicate below. (Select all that apply) |
| Q18: If any of your most recent thyroid lab results were considered too low, indicate below. (Select all that apply) |
| Q19: Have you used eyedrops to treat TED? |
| Q20: How did/do eyedrops affect your symptoms?* |
| Q21: How did/do eyedrops affect your symptoms?* |
| Q22: Have you used an oral steroid (prednisone taken by mouth) to treat TED? |
| Q23: How did/does the oral steroid affect your symptoms?* |
| Q24: How did/does the oral steroid affect your symptoms?* |
| Q25: Have you used an intravenous (IV) steroid (prednisolone administered through your vein) to treat TED? |
| Q26: How did/does the IV steroid affect your symptoms?* |
| Q27: How did/does the IV steroid affect your symptoms?* |
| Q28: Have you used selenium to treat TED? |
| Q29: How did/does the selenium affect your symptoms?* |
| Q30: How did/does the selenium affect your symptoms?* |
| Q31: Have you used Actemra^®^ (tocilizumab) to treat TED? |
| Q32: How did/does Actemra^®^ (tocilizumab) affect your symptoms?* |
| Q33: How did/does Actemra^®^ (tocilizumab) affect your symptoms?* |
| Q34: Have you used CellCept^®^ (mycophenolate mofetil) to treat TED? |
| Q35: How did/does CellCept^®^ (mycophenolate mofetil) affect your symptoms?* |
| Q36: How did/does CellCept^®^ (mycophenolate mofetil) affect your symptoms?* |
| Q37: Have you used Rituxan^®^ (rituximab) to treat TED? |
| Q38: How did/does Rituxan^®^ (rituximab) affect your symptoms?* |
| Q39: How did/does Rituxan^®^ (rituximab) affect your symptoms?* |
| Q40: Have you used Tepezza^®^ (teprotumumab-trbw) to treat TED? |
| Q41: How did/does Tepezza^®^ (teprotumumab-trbw) affect your symptoms?* |
| Q42: How did/does Tepezza^®^ (teprotumumab-trbw) affect your symptoms?* |
| Q43: Have you had orbital radiation to treat TED? |
| Q44: How did/does orbital radiation affect your symptoms?* |
| Q45: How did/does orbital radiation affect your symptoms?* |
| Q46: Have you had orbital decompression surgery (operation to remove fat and bone from around your eye) to treat TED? |
| Q47: How did orbital decompression surgery affect your symptoms?* |
| Q48: How did orbital decompression surgery affect your symptoms?* |
| Q49: Have you had strabismus surgery (operation on your eye muscles to straighten out your eyes) to treat TED? |
| Q50: How did strabismus (eye muscle) surgery affect your symptoms?* |
| Q51: How did strabismus (eye muscle) surgery affect your symptoms?* |
| Q52: Have you had eyelid repair surgery to treat TED? |
| Q53: How did eyelid repair surgery affect your symptoms?* |
| Q54: How did eyelid repair surgery affect your symptoms?* |
| Q55: Which physical symptoms of TED have bothered you the most (made life more difficult/caused pain or suffering) over the past 2 months? |
| Q56: Over the past 2 months, which activities have been made easier by medication(s) or orbital radiation? |
| Q57: Over the past 2 months, which activities have been made easier by surgery? |
| Q58: If possible, which treatment(s) would you like your doctor to implement? |
| Q59. What is the reason for your choice? (Free Form) |
| Q60: Do you feel that your health coverage/insurance and/or the cost of treatment in any way limit your TED medical care? |
| Q61. Is there a reason that you may not be able to have the TED treatment of your choice? If so, please tell us here. (Free Form) |
| Q62. Is there anything else you'd like to tell us about being diagnosed with or living with TED? (Free Form) |

*Queries about experiences with medications and surgery were expanded to 2 questions each to cover a larger range of symptoms.

TED, thyroid eye disease.

**SUPPLEMENTAL TABLE 2** **Narrative responses regarding obtaining a diagnosis.**

| - “I live in [*country in Africa*] … it has been difficult because I was misdiagnosed for over 1 year. My marriage suffered because we both did not know what was wrong with me.” |
| --- |
| - “It took 2 eye doctors to finally get a diagnosis, but it seems like not enough is known about it.” |
| - “Almost went blind because of missed Dx by so-called ophthalmologists. I asked several MDs if this could be TED and was told NO. Should be consequences for doctors misdiagnosed and saying it’s just blepharitis and go home.” |
| - “Took several doctors gaslighting me before a neuro-ophthalmologist finally did [diagnose].” |
| - “I went to my primary care physician and was sent to a gastroenterologist. My husband tore our master bath down to the studs looking for hidden mold (there wasn't any). I saw an allergist that treated me with antibiotics and did a CAT scan of my sinuses. I excluded all cosmetic and health and beauty products and tried various exclusion diets in search of an allergy. Finally, the second allergist I saw thought my neck looked large and thought the cause might be my thyroid. His office arranged for lab work and I was diagnosed with severe Graves' disease and TED. I believe he saved my life.” |
| - “I had no idea about TED (disease/symptoms) until I saw my endocrinologist. She needed this evaluation to determine if I was a candidate for iodine treatment. I've always been diagnosed with "dry eye syndrome", so I just thought that was causing my symptoms.” |
| - “I went to 3 highly recommended eye doctors and none of them diagnosed me with TED or thyroid problems.” |

CAT, computed axial tomography; Dx, diagnosis; TED, thyroid eye disease.

**SUPPLEMENTAL TABLE 3 Narrative responses regarding specific symptoms.**

| *Eyelids:*   - “Now that the Graves’ in my eyes is no longer active my eyelids are too droopy, they often will flip under when washing my face.” - “Eyelid was lowered too much during eyelid surgery. I still can't squint.” - “I am left with very heavy eyelids and fat prolapse.” - “Drooping lids cause eyes to tire. Eyelashes touch pupils irritating them.” - “My right eyelid looks droopy. The watering of the eyes are not fun to deal with and the pains in my eyes hurt badly too.” |
| --- |
| *Eyebrow area:*   - “My eyebrow is up all the time so, other than looking like monstrous, I also look like angry all the time.” - “The swelling under my brows is unbearable.” |
| *Bulging:*   - “I do hate the way 1 bulges more than the other.” - “My eyelids still do not close, even after 4 surgeries! My eyes water terribly every night while sleeping bc they do not close all the way.” |
| *Double vision:*   - “The horrible double vision was very disorienting.” - “I still have double vision when looking to the left and right.” - “Even with prism glasses, I'm still limited in the angles I can look without incurring double vision.” |
| *Headache/migraine/pain:*   - “I have had near constant occipital pain and migraines because of cocking my head to look up at monitors other people and movie screens etc.” - “Some days I can’t even have my eyes open due to pain and sensitivity and have to sit in a dark room with my eyes closed.” - “I'm not sure if my current pain is due to a reactivation of TED or something else. I've had worsening headaches centered around my sinuses/eyes.” - “I suffer a lot from eye pain, headaches, and light sensitivity. Is it possible that this is the reason I've had worsening headaches centered around my sinuses/eyes? It isn't on their radar.” |
| *Blurred vision*   - “Blurred vision has had the biggest negative impact on me.” - “Have slight blurred vision for distance.” |
| *Multiple symptoms:*   - “Post-surgical … everything has improved … but occasionally my eyes will still bulge, puff, water (albeit without the old grittiness and pain) and feel some pressure from time to time, esp or coincidentally in response to perceived traumatic or highly emotional times.” - “Drooping eyelids make it difficult to see & perhaps my dry eye wetness will allow me to wear eye makeup again and stop always wiping my eyes with a handkerchief. Wet eyes are a nuisance & all this … are still YEARS after I’m better! (Means no more double vision or severe eye bulging any longer).” - “Affecting everything with me, losing my sight for almost 3 years, and in pain until remission…” - “It's good days and not so good but the swollen lid & 1 eye seems to look inward more and still bulgy & occasional bothersome irritation.” - “TED [delivered] brutal symptoms as I desperately searched for answers for nearly 2 years … terrible facial and eye pain while my eyes swelled up and began to slightly bulge.” - “I still have dry eyes, eyelid retraction though minimal, and my eyelid does not close all the way during sleep causing dry eye. Also have sensitivity to light.” - “I'm experiencing dry eye and resulting pain and now I have inflamed oil glands on my eyelids, which is just another time-consuming health issue.” - “I … need to pause to wipe my eyes while trying to write or read; also, I hate the eyelid swelling but wouldn't want aggressive treatment.” - “I had a total thyroidectomy at age 15 for toxic goiter/Graves’. In my 20s, only my right eye was affected by bulging out. IV steroids took care of it. Now 45 years later, I have pain and stiffness in the muscles behind my eyes when trying to focus.” - “It started with right eye eyelid swelling, then left eyelid swelling, and then 3 months of misdiagnosis, terrible bulging, muscle pain, and palpitation.” |
| *Stress and symptoms:*   - “Stress is the major cause of my TED symptoms and work is stressful.” - “Occasionally my eyes will still bulge, puff, water (albeit without the old grittiness and pain) and feel some pressure from time to time, esp or coincidentally in response to perceived traumatic or highly emotional times.” |

bc, because; esp, especially; IV, intravenous; TED, thyroid eye disease.

**SUPPLEMENTAL TABLE 4** **Narrative responses regarding the general experience of TED.**

| - “Awful disease, much misunderstood in terms of effect on quality of life.” |
| --- |
| - “It is not the worst thing in the world but I'd sure like to get back to normal!” |
| - “It has affected all aspects of my life ... a very frustrating disease.” |
| - “It’s scary and I wish I had better care.” |
| - “It has totally changed my life in so many ways.” |
| - “I just can’t wait to feel like myself again.” |
| - “It sucks and I have “lost my pretty.” |
| - “I hate it. I wish I knew why I got it. I was pretty before, I had pretty eyes. And now I’m different looking. My specialist doesn’t care. It’s hard to explain to strangers life before Graves.” |

TED, thyroid eye disease.
